# Personalizing Prediction of High Opioid Use in the Neurointensive Care Unit: Development and Validation of a Stratified Risk Model for Acute Brain Injury Due to Stroke or Traumatic Brain Injury

**DOI:** 10.3390/jcm13237055

**Published:** 2024-11-22

**Authors:** Wei Yun Wang, Ian C. Holland, Christine T. Fong, Samuel N. Blacker, Abhijit V. Lele

**Affiliations:** 1 Department of Anesthesiology and Pain Medicine, Harborview Medical Center, University of Washington, Seattle, WA 98104, USA; weiyun@uw.edu (W.Y.W.); cfong@uw.edu (C.T.F.); 2 School of Medicine, University of Washington, Seattle, WA 98104, USA; hollandi@uw.edu; 3 Department of Anesthesiology, University of North Carolina, Chapel Hill, NC 27599, USA; samuel_blacker@med.unc.edu

**Keywords:** opioids, neurocritical care, ischemic stroke, subarachnoid hemorrhage, intracerebral hemorrhage, traumatic brain injury, prediction, personalized, model, calculator

## Abstract

**Background/Objectives:** This study aimed to develop and validate a stratified risk model for predicting high opioid use in patients with acute brain injury due to stroke or traumatic brain injury (TBI) admitted to a neurocritical care intensive care unit. **Methods**: We examined the factors associated with the use of high-opioids (≥75th quartile, ≥17.5 oral morphine equivalent/ICU day) in a retrospective cohort study including patients with acute ischemic stroke, spontaneous intracerebral hemorrhage, spontaneous subarachnoid hemorrhage, and TBI. We then developed, trained, and validated a risk model to predict high-dose opioids. **Results**: Among 2490 patients aged 45–64 years (*β* = −0.25), aged 65–80 years (*β* = −0.97), and aged ≥80 years (*β* = −1.17), a history of anxiety/depression (*β* = 0.57), a history of illicit drug use (*β* = 0.79), admission diagnosis (*β* = 1.21), lowest Glasgow Coma Scale Score (GCSL) [GCSL 3–8 (*β* = −0.90], {GCS L 9–12 ((*β* = −0.34)], mechanical ventilation (*β* = 1.21), intracranial pressure monitoring (*β* = 0.69), craniotomy/craniectomy (*β* = 0.6), and paroxysmal sympathetic hyperactivity (*β* = 1.12) were found to be significant predictors of high-dose opioid use. When validated, the model demonstrated an area under the curve ranging from 0.72 to 0.82, accuracy ranging from 0.68 to 0.91, precision ranging from 0.71 to 0.94, recall ranging from 0.75 to 1, and F1 ranging from 0.74 to 0.95. **Conclusions**: A personalized stratified risk model may allow clinicians to predict the risk of high opioid use in patients with acute brain injury due to stroke or TBI. Findings need validation in multi-center cohorts.

## 1. Introduction

The management of pain in the neuro intensive care unit (neuro ICU) is a critical challenge, particularly for patients admitted with acute ischemic stroke (AIS), spontaneous intracerebral hemorrhage (s-ICH), spontaneous subarachnoid hemorrhage (SAH), and traumatic brain injury (TBI) [1,2]. These conditions often require aggressive treatment strategies, including sedation and pain management, to mitigate the effects of elevated intracranial pressure, autonomic dysregulation, and other complications. Opioids are a mainstay of pain management in the ICU; however, their use may be associated with significant risks, including respiratory depression, delirium, and prolonged ICU stays. The prevalence of opioid use in the neuro ICU may vary widely depending on the diagnosis [3,4,5,6,7,8,9,10,11,12], severity of illness, presence of comorbidities [13], and institutional practices [5], making it a critical area of study.

Opioid use in critical care settings is well-documented [14], especially in those with intracranial pressure monitoring [15,16] and in those with paroxysmal sympathetic hyperactivity (PSH) [17], where established guidelines for managing pain, agitation, and delirium (PAD) [18] recommend considering non-opioid adjuncts. However, this guideline is not specific to the neurocritical care population, where patients’ pathophysiology and clinical course can differ significantly from other ICU patients. Studies have shown that patients with neurological injuries who are treated with opioids have a higher propensity for neurological and non-neurological adverse effects, such as constipation, ileus, and delirium, as well as infectious complications such as Clostridium difficile infections [19] and risk of continued use or risk of opioid use disorder.

Despite the critical importance of managing pain in the neuro ICU, there is a significant gap in understanding the specific predictors of high opioid use in this population. The effect of the type of brain injury on the selection of opioids is not fully understood. Existing studies on opioid use do not stratify patients by neurological diagnosis or account for the unique challenges posed by conditions such as AIS, s-ICH, SAH, and TBI. Consequently, there is a lack of tailored prediction models that can guide clinicians in determining which patients are at the highest risk of requiring high doses of opioids and who may benefit from alternative pain management strategies.

To address these gaps, this study aimed to develop and validate a set of prediction models for high opioid use across four major neurological diagnostic groups: AIS, s-ICH, SAH, and TBI. The primary objective was to identify key predictors of high opioid use, defined as requiring ≥17.5 Oral Morphine Equivalents (OME) per day during their ICU stay, and to validate these models in a large cohort of neuro ICU patients. Additionally, we aimed to implement these models in an online calculator to facilitate personalized pain management strategies in clinical practice. By stratifying patients into low, moderate, and high-risk categories, this tool could help clinicians identify those at risk of opioid-related complications and guide decision-making.

## 2. Materials and Methods

### 2.1. Institutional Review Board Review and Approval

This study (#18543) was reviewed by the University of Washington’s Institutional Review Board and approved on 9 April 2024. Due to the retrospective study design, a waiver of consent was granted.

### 2.2. Study Design, Population, and Clinical Setting

This retrospective observational study was conducted in the neurointensive care unit of a single tertiary care center. The study population consisted of 2490 patients, admitted between January 2021 and December 2023 with a primary diagnosis of acute ischemic stroke (AIS), intracerebral hemorrhage (s-ICH), subarachnoid hemorrhage (SAH), or traumatic brain injury (TBI). Patients were included if they were 18 years or older and had complete data for the variables of interest. This study was conducted in a 413-bed academic medical center, a Level I trauma center, and a comprehensive stroke center. Notably, no specific institutional protocols were in place for prescribing opioids to patients in the neurocritical care unit. The attending neurointensivist made these decisions at their discretion, based on individual patient assessments and clinical judgment. The absence of specific protocols allowed for a real-world exploration of clinical practices.

### 2.3. Data Collection and Curation

Data were extracted from electronic medical records, including demographic information, clinical characteristics, treatment variables, and outcomes. Key variables included age, admission diagnosis, pre-admission anxiety/depression, illicit drug use (based on Elixhauser comorbidity groups), placement of intracranial pressure (ICP) monitor, performance of a craniotomy/craniectomy, and the presence of paroxysmal sympathetic hyperactivity (PSH). The primary outcome was high opioid use, defined as requiring ≥17.5 oral morphine equivalents (OMEs) per day during the ICU stay.

We calculated the OME per day of ICU stay for each patient and determined the distribution of daily opioid use. The 75th percentile (quartile) of opioid use in our dataset was ≥17.5 OME/day, which we defined as “high opioid use” for the purposes of this study. While this threshold may differ from other studies, it reflects the population characteristics and prescribing practices observed in our neuro ICU. Data curation involved preprocessing to handle missing values using median imputation for continuous variables and mode imputation for categorical variables, using the preProcess function from the caret package in RStudio (version 4.0.2) [20]. The continuous variables were checked for normality, and the categorical variables were coded as binary indicators. The dataset was then randomly split into training (70%) and validation (30%) cohorts using stratified sampling to ensure representation across all diagnostic groups.

### 2.4. Model Development

Predictive models for high opioid use were developed for each diagnostic group (AIS, s-ICH, SAH, and TBI) using logistic regression with elastic net regularization. The elastic net model was chosen to handle potential multicollinearity among predictors and to ensure model generalizability. The glmnet package (version 4.1-1) [21] was used to perform the regularization, with the optimal lambda (λ) selected based on the lowest cross-validation error.

### 2.5. Multicollinearity Assessment

The Variance Inflation Factor (VIF) assessed the predictors’ multiplicity. A VIF value ≥ 10 was considered indicative of multicollinearity. No predictors exceeded this threshold, indicating the fact that multicollinearity did not compromise the models.

### 2.6. Model Validation

Model performance was assessed on both the training and validation cohorts. Key performance metrics included the area under the curve (AUC), which was evaluated using the pROC package (version 1.17.0.1) [22] to assess the discriminative ability of the models. Accuracy, precision, recall, and F1 score were calculated using the caret package to provide a comprehensive view of model performance. Calibration plots were generated using the rms package (version 6.2-0) [23] to assess the agreement between predicted probabilities and observed outcomes.

### 2.7. Feature Selection and Multicollinearity

Stepwise feature selection was performed using the stepAIC function from the MASS package (version 7.3-53) [24] to identify the most significant predictors. Variance Inflation Factor (VIF) values were calculated using the car package (version 3.0-10) [25] to assess multicollinearity among predictors. VIF values below ten were considered acceptable, indicating that multicollinearity was not a significant issue.

### 2.8. Online Calculator Development

Based on the final models, an online risk calculator was developed to enable clinicians to quickly assess the risk of high opioid use in individual patients. The calculator stratifies patients into low, moderate, and high-risk categories based on their predicted probability of requiring ≥17.5 OME/day. The calculator is accessible online [26], and its design allows easy updates as new data become available.

## 3. Results

### 3.1. Derivation of the Study Cohort

**Figure 1:**
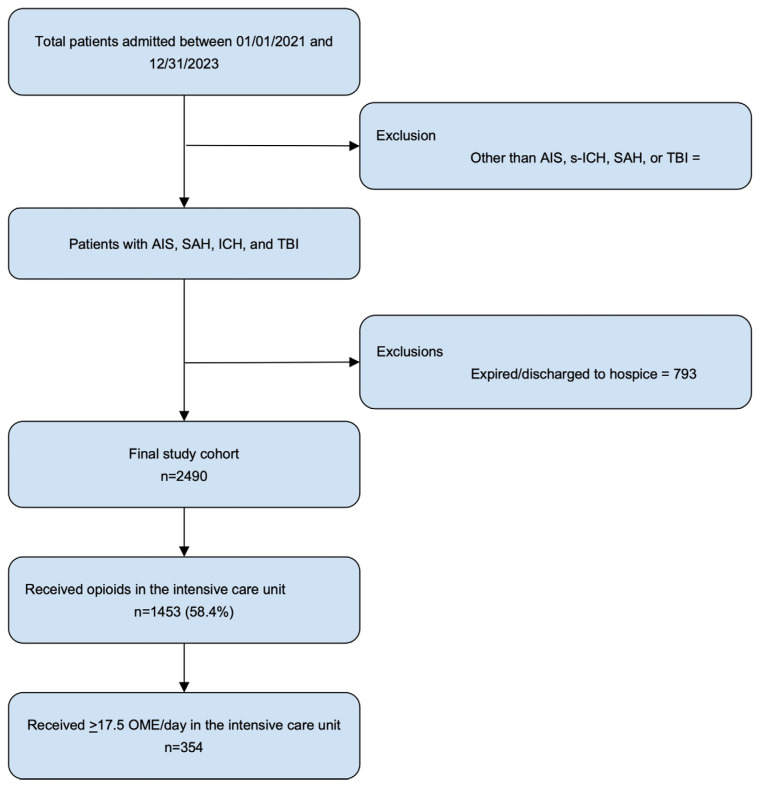
The creation of the study sample. Abbreviations: AIS: acute ischemic stroke; s-ICH: spontaneous intracerebral hemorrhage; SAH: spontaneous subarachnoid hemorrhage; TBI: traumatic brain injury; OME: oral morphine equivalent. Note: the patients discharged to hospice or who expired were excluded to avoid confounding outcomes unrelated to opioid use, as these patients often had poor prognoses independent of pain management strategies, and opioids are commonly used in end-of-life care.

Figure 1 highlights the creation of the study sample.

### 3.2. Study Sample Characteristics

The final study sample comprised 2490 patients. The patients had a median age of 62 years [IQR 18–101], with 59.8% (n = 1488) being males. Among them, 22.8% (n = 568) had AIS, 13.4% (n = 333) had SAH, 15.5% (n = 387) had s-ICH, and 48.3% (n = 1202) had TBI. In addition, 35.8% (n = 891) were mechanically ventilated and 14.8% (n = 369) had an ICP monitor placed. Out of 2490 patients, opioids were administered to 58.35% (n = 1453). Notably, 30% of patients with AIS (n = 171), 44% with s-ICH (169), 83% with SAH (n = 275), and 70% with TBI (n = 838) received opioids in the ICU. Table 1 compares the groups of patients that received opioids and those that did not.

A list of medications to treat pain, agitation, and distress amongst those that received opioids and those that did not are included in Table 2.

Amongst patients receiving opioids, we further categorized patients with high OME use/day of ICU stay into those receiving ≥17.5 OME and those with <17.5 OME. We examined the association between high OME use and infectious and non-infectious complications to understand the importance of classifying patients into high and low OME use. Table 3 compares the two groups.

### 3.3. Association Between High Opioid Use and Infections and Non-Infectious Complications and Use of Medications to Counteract Adverse Events

To describe the importance of classifying patients into high-opioid-use and non-opioid-use groups, we examined infectious and non-infectious complications associated with high opioid use and presented them in Table 4.

### 3.4. Creating a Model to Predict High-Dose Opioid Use

We reviewed the results of the multivariable analysis (Table 3) to inform the prediction model. The following variables were chosen for the prediction model: age, diagnosis, history of anxiety/depression, illicit substance use, lowest Glasgow Coma Scale score, paroxysmal sympathetic hyperactivity, intracranial pressure monitoring, and craniotomy/craniectomy. The model was then tested in the training cohort. The β coefficients of the models provide insights into the relationships between predictors and the likelihood of high-dose opioid use, presented in Table 5.

### 3.5. Model Performance in the Training Cohort

The model was then tested in the training cohort. Its performance is presented in Table 6.

### 3.6. Validation of Model

The model was then validated, and the results are shown in Table 7.

Model Performance in the Validation Cohort

Traumatic brain injury (TBI): in the validation cohort, the metrics were consistent with the training performance, indicating that the model generalizes well to unseen data.

Subarachnoid hemorrhage (SAH): the SAH model’s validation results are slightly lower than its training performance, indicating good generalization, with minimal overfitting.

Acute ischemic stroke (AIS): the AIS model maintained strong validation performance.

The consistent results between training and validation cohorts suggest that the model is robust and generalizes well.

Intracerebral hemorrhage (s-ICH): the s-ICH model’s validation metrics included a high recall, which indicates that the model is particularly effective in identifying true positive cases of high opioid use.

### 3.7. Online Calculator

Based on the final validated models, an online calculator was developed to facilitate the prediction of high opioid use in neuro ICU patients. This calculator stratifies patients into low-, moderate-, and high-risk categories based on the likelihood of requiring ≥17.5 OME/ICU day. The output includes both a risk category and a percentage risk, providing clinicians with actionable insights for personalized opioid management. The calculator is accessible via the following link: [https://nccs.shinyapps.io/nccalculator/] (accessed on 1 September 2024).

## 4. Discussion

This study advances neurocritical care by developing and validating prediction models for high opioid use across four major neurological diagnostic groups: AIS, s-ICH, SAH, and TBI. The models demonstrated strong performance, with AUC values ranging from 0.72 to 0.82. The development of an online calculator based on these models allowed for the real-time application of these findings in clinical practice, offering a personalized approach to opioid management in the Neuro ICU.

This study’s findings underscore the importance of tailoring opioid management strategies to the specific needs of different neurological conditions. The significant coefficients for age, pre-admission anxiety/depression, illicit drug use, mechanical ventilation, ICP monitoring, craniotomy/craniectomy, and PSH across all diagnostic groups highlight the complex interplay between these factors and opioid requirements. Younger age consistently emerged as a predictor of higher opioid use, potentially reflecting differences in pain sensitivity, metabolic demands, and prescribing patterns compared to older populations. The model for s-ICH demonstrated the highest AUC, suggesting that patients with this diagnosis may have more predictable opioid requirements compared to other groups. The differences in model performance likely reflect variations in underlying pathophysiology, treatment approaches, and patient demographics between TBI and CVA populations, as well as the heterogeneity of clinical care in TBI patients.

### 4.1. Implications for Clinical Practice

The implementation of these models into an online calculator represents a significant step forward in personalizing opioid management in the neuro ICU. The high-dose opioid risk calculator is designed as a decision-support tool to stratify patients by their likelihood of requiring high opioid doses (≥17.5 OME/day). While pain management is individualized, the calculator helps identify patients at risk for opioid-related complications and suggests alternative strategies. It does not replace clinical judgment but complements it by highlighting risk factors and encouraging evidence-based, patient-centered care. By providing clinicians with an easy-to-use tool that stratifies patients into risk categories, the calculator can help guide decisions regarding opioid dosing and alternative pain management strategies. This is particularly important given the risks associated with high opioid use, including respiratory depression, delirium, and prolonged ICU stays. Regarding the association between opioids and delirium, there seems to be a propensity towards a higher risk of delirium with the use of meperidine in the perioperative period [27], and there are conflicting data on the significant association between opioids and delirium [28].

Furthermore, the use of these models in clinical practice has the potential to reduce variability in opioid-prescribing practices, ensuring that patients receive the most appropriate level of care based on their risk profile. This approach aligns with the broader goals of personalized medicine, which seeks to tailor treatment to each patient’s unique characteristics. Thus, neurocritical care practitioners may use this online calculator during bedside multidisciplinary rounds. The risk calculated for high opioid use may stimulate consideration of non-opioid adjuncts to allow treatment of pain, agitation, and distress, balancing the expectations and minimizing adverse effects of high-dose opioids for more extended periods in the ICU.

### 4.2. Future Directions and Limitations

While the models developed in this study have shown promising results, further validation in a multi-center cohort is necessary to confirm their generalizability. The current study was conducted in a single tertiary care center, and the patient population may only partially represent the diversity seen in other institutions. Future research should focus on validating these models in different settings and refining them by incorporating additional predictors. Another limitation is this study’s retrospective design, which relies on the accuracy and completeness of existing medical records. Prospective studies provide a more rigorous assessment of the model’s predictive power and impact on clinical outcomes. Finally, developing the online calculator is a significant achievement, but its implementation in clinical practice will require ongoing evaluation and refinement. User feedback and data from real-world use will be crucial in ensuring that the tool remains relevant and effective in guiding opioid management. Future iterations of the calculator will incorporate features such as (1) displaying risk as low, medium, or high with color-coded indicators (green, yellow, red); (2) providing specific warnings about adverse effects of high opioid doses (e.g., respiratory depression, delirium, prolonged ICU stays); (3) suggesting alternative non-opioid therapies; and (4) allowing users to input patient outcomes to refine the model through real-world learning and database expansion.

## 5. Conclusions

The prediction models developed in this study provide a valuable tool for personalizing opioid management in the neuro ICU. By identifying patients at risk for high opioid use, these models can help clinicians tailor their treatment strategies, potentially improving patient outcomes and reducing the incidence of opioid-related complications. The online calculator derived from these models is essential to integrating personalized medicine into neurocritical care, offering a practical solution for real-time decision-making. Future validation in multi-center cohorts and continued refinement of the models will be vital in maximizing their clinical utility.

## Figures and Tables

**Table 1:** Comparison of demographic and clinical characteristics between patients who received opioids and those who did not.

	No Opioids	Opioids	Univariable Analysis Unadjusted Odds Ratio [95% CI]	Multivariable Analysis Adjusted Odds Ratio [95% CI]
	N = 1037	N = 1453		
Age				
18–44 years	138 (13.3%)	393 (27.0%)	Reference	Reference
45–64 years	274 (26.4%)	544 (37.4%)	0.70 [0.55;0.89]	0.97 [0.73;1.29]
65–79 years	421 (40.6%)	397 (27.3%)	0.33 [0.26;0.42]	0.56 [0.42;0.76]
≥80 years	204 (19.7%)	119 (8.19%)	0.21 [0.15;0.28]	0.43 [0.30;0.62]
Sex				
Female	397 (38.3%)	605 (41.6%)	Reference	
Male	640 (61.7%)	848 (58.4%)	0.87 [0.74;1.02]	Excluded
Race/ethnicity				Excluded
White	796 (76.8%)	1118(76.9%)	Reference	
Black or African-American	64 (6.2%)	109 (7.5%)	1.21 [0.88;1.67]	
Asian	88 (8.5%)	115 (7.9%)	0.93 [0.69;1.24]	
Other	89 (8.58%)	111 (7.64%)	0.88 [0.66;1.19]	
Language				
English	901 (87.0%)	1285 (88.4%)	Reference	
Non-English	135 (13.0%)	168 (11.6%)	0.87 [0.69;1.11]	Excluded
Insurance carrier				
Commercial	226 (21.8%)	430 (29.6%)	Reference	
Non-commercial	811 (78.2%)	1023 (70.4%)	0.66 [0.55;0.80]	0.94 [0.73;1.21]
Medicaid	160 (15.43%)	390 (26.84%)	1.28 [1.00;1.64]	
Medicare	585 (56.41%)	533 (3668%)	0.47 [0.29;0.58]	
Self-pay	24 (2.31%)	27 (1.86%)	0.59 [0.33;1.04]	
Tricare	9 (0.87%)	12 (0.83%)	0.70 [0.29;1.69]	
Worker’s compensation	6 (0.58%)	29 (2%)	2.54 [1.04;6.28]	
Other	27 (2.6%)	32 (2.2%)	0.62 [0.36;1.07]	
Elixhauser comorbidity				
Alcohol abuse	124 (12.0%)	262 (18.0%)	1.62 [1.29;2.04]	1.06 [0.80;1.40]
Tumor/Cancer	128 (12.3%)	151 (10.4%)	0.82 [0.64;1.06]	Excluded
Anxiety-depression	223 (21.5%)	397 (27.3%)	1.37 [1.14;1.66]	1.63 [1.30;2.03]
Psychoses	77 (7.43%)	127 (8.74%)	1.19 [0.89;1.61]	Excluded
Substance use disorder	71 (6.85%)	198 (13.6%)	2.14 [1.62;2.86]	1.33 [0.94;1.89]
Chronic pain	177 (17.1%)	266 (18.3%)	1.09 [0.88;1.34]	Excluded
Pre-admission OME	1.48 (9.89)	2.58 (17.1)	1.01 [1.00;1.01]	Excluded
Admitting diagnosis				
Acute ischemic stroke	397 (69.9%))	171 (30.11%)	Reference	Reference
Intracerebral hemorrhage	218 (56.33%)	169 (43.67%)	1.80 [1.37;2.36]	1.18 [0.86;1.61]
Subarachnoid hemorrhage	58 (17.42%)	275 (82.58%)	11.0 [7.88;15.4]	5.85 [3.98;8.60]
Traumatic brain injury	364 (30.28%)	838 (69.72%)	5.34 [4.30;6.65]	4.68 [3.64;6.01]
Admission Glasgow Coma Scale score				
13–15	797 (76.9%)	882 (60.7%)	Reference	Reference
3–8	118 (11.4%)	441 (30.4%)	3.37 [2.70;4.24]	0.93 [0.62;1.40]
9–12	122 (11.8%)	130 (8.95%)	0.96 [0.74;1.26]	0.86 [0.63;1.18]
Mechanical ventilation	216 (20.8%)	675 (46.5%)	3.29 [2.75;3.96]	2.13 [1.45;3.11]
Maximum CIWA score	11.0 (8.95)	12.1 (7.76)	1.02 [0.99;1.05]	Excluded
Paroxysmal sympathetic hyperactivity	22 (2.1%)	163 (11.22%)	5.83 [3.71;9.17]	2.53 [1.45; 4.32]
Intracranial pressure monitoring	24 (2.31%)	345 (23.7%)	13.1 [8.74;20.5]	5.50 [3.44;8.80]
Lowest Admission Glasgow Coma Scale score in the ICU				
13–15	596 (57.5%)	597 (41.1%)	Reference	Reference
3–8	254 (24.5%)	700 (48.2%)	2.75 [2.29;3.31]	0.81 [0.55;1.21]
9–12	187 (18.0%)	156 (10.7%)	0.83 [0.65;1.06]	0.80 [0.62;1.18]
Gastrostomy feeding tube	114 (11.0%)	295 (20.3%)	2.06 [1.64;2.61]	1.19 [0.84;1.69]
Tracheostomy	10 (0.96%)	85 (5.85%)	6.29 [3.41;13.0]	1.46 [0.66;3.23]
Craniotomy/craniectomy	108 (10.4%)	527 (36.3%)	4.89 [3.91;6.15]	2.21 [1.71;2.87]

Abbreviations: ICU: intensive care unit; OME: oral morphine equivalent; CIWA: Clinical Institute Withdrawal Assessment of Alcohol Scale.

**Table 2:** Medications used to treat pain, agitation, and distress in patients with acute brain injury.

	No Opioids	Opioids	Overall
	(N = 1037)	(N = 1453)	(N = 2490)
Propofol	197 (19.0%)	650 (44.7%)	847 (34.0%)
Ketamine	6 (0.6%)	50 (3.4%)	56 (2.2%)
Dexmedetomidine	46 (4.4%)	254 (17.5%)	300 (12.0%)
Phenobarbital	23 (2.2%)	58 (4.0%)	81 (3.3%)
Gabapentin	152 (14.7%)	539 (37.1%)	691 (27.8%)
Acetaminophen	846 (81.6%)	1421 (97.8%)	2267 (91.0%)
Haloperidol	65 (6.3%)	170 (11.7%)	235 (9.4%)
Quetiapine	109 (10.5%)	321 (22.1%)	430 (17.3%)
Lorazepam	163 (15.7%)	319 (22.0%)	482 (19.4%)
Diazepam	28 (2.7%)	61 (4.2%)	89 (3.6%)
Midazolam	119 (11.5%)	492 (33.9%)	611 (24.5%)

**Table 3:** Comparison of demographic and clinical characteristics between patients who received ≥ 17 OME/day and those receiving < 17 OME/day of ICU stay.

	Daily OME <17.5/ICU Day	Daily OME ≥17.5/ICU Day	Univariable Analysis Unadjusted Odds Ratio [95% CI]	Multivariable Analysis Adjusted Odds Ratio [95% CI]
	N = 1089	N = 364		
Age				
18–44 years	238 (21.9%)	155 (42.6%)	Reference	Reference
45–64 years	410 (37.6%)	134 (36.8%)	0.50 [0.38;0.67]	0.56 [0.41;0.77]
65–80 years	332 (30.5%)	65 (17.9%)	0.30 [0.21;0.42]	0.44 [0.3;0.64]
>80 years	109 (10.0%)	10 (2.75%)	0.14 [0.07;0.27]	0.25 [0.12;0.51]
Sex				
Female	460 (42.2%)	145 (39.8%)	Reference	Reference
Male	629 (57.8%)	219 (60.2%)	1.10 [0.87;1.41]	Excluded
Race/Ethnicity				
Non-White	244 (22.4%)	91 (25.0%)	Reference	Reference
White	845 (77.6%)	273 (75.0%)	0.87 [0.66;1.15]	Excluded
Language				
English	959 (88.1%)	326 (89.6%)	Reference	Reference
Non-English	130 (11.9%)	38 (10.4%)	0.86 [0.58;1.25]	Excluded
Insurance				
Commercial	306 (28.1%)	124 (34.1%)	Reference	Reference
Non-commercial	783 (71.9%)	240 (65.9%)	0.76 [0.59;0.98]	Excluded
Elixhauser comorbidity				
Alcohol abuse	170 (15.6%)	92 (25.3%)	1.83 [1.37;2.43]	Excluded
Tumor/Cancer	114 (10.5%)	37 (10.2%)	0.97 [0.65;1.42]	Excluded
Anxiety/depression	300 (27.5%)	97 (26.6%)	0.96 [0.73;1.25]	Excluded
Psychoses	82 (7.53%)	45 (12.4%)	1.73 [1.17;2.54]	Excluded
Substance use disorder	118 (10.8%)	80 (22.0%)	2.32 [1.69;3.17]	1.89 [1.31;2.73]
Chronic pain	186 (17.1%)	80 (22.0%)	1.37 [1.02;1.83]	1.94 [1.39;2.72]
Pre-admission OME	1.84 (11.9)	4.78 (27.1)	1.01 [1.00;1.02]	Excluded
Admitting diagnosis				
Acute ischemic stroke	151 (13.9%)	20 (5.49%)	Reference	Reference
Intracerebral hemorrhage	147 (13.5%)	22 (6.04%)	1.13 [0.59;2.18]	0.7 [0.35;1.4]
Subarachnoid hemorrhage	196 (18.0%)	79 (21.7%)	3.02 [1.80;5.29]	2 [1.1;3.62]
Traumatic brain injury	595 (54.6%)	243 (66.8%)	3.06 [1.92;5.14]	2.27 [1.34;3.84]
Admission Glasgow Coma Scale score				
13–15	686 (63.0%)	196 (53.8%)	Reference	Reference
3–8	295 (27.1%)	146 (40.1%)	1.73 [1.34;2.23]	1.38 [0.88;2.17]
9–12	108 (9.92%)	22 (6.04%)	0.72 [0.43;1.14]	0.69 [0.38;1.24]
Mechanical ventilation	474 (43.5%)	201 (55.2%)	1.60 [1.26;2.03]	1.7 [0.97;2.97]
Maximum CIWA score	11.6 (7.47)	13.2 (8.30)	1.03 [0.99;1.06]	Excluded
Paroxysmal sympathetic hyperactivity	0.08 (0.27)	0.21 (0.41)	3.04 [2.18;4.25]	2.41 [1.59;3.65]
Intracranial pressure monitoring	223 (20.5%)	122 (33.5%)	1.96 [1.50;2.54]	1.73 [1.21;2.46]
Lowest Admission Glasgow Coma Scale score in the ICU				
13–15	459 (42.1%)	138 (37.9%)	Reference	Reference
3–8	500 (45.9%)	200 (54.9%)	1.33 [1.04;1.71]	0.51 [0.28;0.94]
9–12	130 (11.9%)	26 (7.14%)	0.67 [0.41;1.05]	0.7 [0.41;1.21]
Gastrostomy feeding tube	212 (19.5%)	83 (22.8%)	1.22 [0.91;1.63]	0.75 [0.48;1.16]
Tracheostomy	52 (4.78%)	33 (9.07%)	1.99 [1.25;3.12]	1.05 [0.57;1.93]
Craniotomy/craniectomy	365 (33.5%)	162 (44.5%)	1.59 [1.25;2.03]	1.25 [0.95;1.65]

**Table 4:** Univariable analysis of infections, non-infectious complications, and adjunct medication administration for pain, agitation, and distress in patients receiving high-dose opioids.

Condition/Medication	Patients Receiving <17.5 OME/ICU Day N = 1089	Patients Receiving ≥17.5 OME/ICU Day N = 364	Unadjusted Odds Ratio [95% CI]
Non-infectious complications			
Code blue	14 (1.3%)	10 (2.7%)	2.17 [0.95;4.93]
Rapid response	265 (24%)	83 (23%)	0.92 [0.69;1.22]
Delirium	19 (1.7%)	4 (1.1%)	1.08 [0.85;1.37]
Ileus	83 (7.6%)	40 (11%)	1.50 [1.00;2.23]
Venous thromboembolism	39 (3.6%)	18 (4.9%)	1.40 [0.79;2.48]
One or more out-of-operating room non-procedural intubation	42 (88%)	24 (92%)	1.76 [1.05;2.95]
Infectious complications			
Ventilator-associated pneumonia (VAP)	53 (4.9%)	41 (11%)	2.48 [1.62;3.80]
Catheter-associated urinary tract infections (CAUTI)	19 (1.7%)	4 (1.1%)	0.63 [0.21;1.85]
Clostridium difficile infection	18 (1.7%)	7 (1.9%)	1.17 [0.48;2.82]
Medications to counteract adverse events			
Naloxegol	10 (0.9%)	32 (8.8%)	10.40 [5.06;21.38]
Naloxone	20 (1.8%)	17 (4.7%)	2.62 [1.36;5.06]

**Table 5:** Beta coefficients of the model to predict high-dose opioid use.

Variables	Overall	AIS	s-ICH	SAH	TBI
Intercept	−2.73	−2.38	−2.58	−0.087	−0.51
Age					
18–44 years	Reference	Reference	Reference	Reference	Reference
45–64 years	−0.25	−0.59	−1.10	−0.89	−0.33
65–80 years	−0.97	−0.28	−1.52	−1.35	−0.94
≥80 years	−1.17	−1.72	−0.69	−2.54	−2.11
History of Anxiety/depression	0.57	0.03	0.63	0.51	−0.03
History of Illicit Substance Use	0.79	0.20	0.45	0.89	0.69
Lowest Glasgow Coma Scale score					
13–15	Reference	Reference	Reference	Reference	Reference
3–8	−0.90	0.55	0.39	−1.50	−0.87
9–12	−0.34	0.12	1.38	−1.15	−0.76
Mechanical ventilation	1.21	0.42	0.47	−0.28	1.01
Craniotomy/craniectomy	0.60	0.11	1.04	1.02	0.26
Intracranial pressure monitoring	0.69	0.43	0.38	1.40	0.75
Paroxysmal sympathetic hyperactivity	1.12	2.35	1.35	1.32	0.45

TBI: Older age groups were associated with lower opioid use, while mechanical ventilation and PSH were strong positive predictors. SAH: the presence of ICP monitoring and craniotomy were significant predictors of high opioid use. AIS: paroxysmal sympathetic hyperactivity had the strongest association with high opioid use, followed by GCS scores. s-ICH: the need for craniotomy and mechanical ventilation were significant predictors, with a robust positive association observed for PSH.

**Table 6:** The performance of the model in the training cohort.

	AIS	s-ICH	SAH	TBI
AUC	0.72	0.80	0.78	0.73
Accuracy	0.90	0.85	0.74	0.72
Precision	0.91	0.87	0.75	0.74
Recall	0.99	0.97	0.85	0.92
F1	0.95	0.92	0.79	0.82

Abbreviations: AUC (area under the curve); AIS (acute ischemic stroke): the VIF values were within acceptable ranges, indicating minimal multicollinearity issues. s-ICH (intracerebral hemorrhage): the VIF values for key predictors were below 5, indicating minimal multicollinearity and suggesting that the model is robust and reliable. SAH (subarachnoid hemorrhage): the VIF values for predictors, such as the lowest GCS (3–8) (VIF = 4.92) and mechanical ventilation (VIF = 4.03) suggested moderate multicollinearity, though it did not significantly impact model robustness. Traumatic brain injury (TBI): the model’s VIF values indicated moderate multicollinearity, particularly for predictors such as the lowest GCS (3–8) (VIF = 5.75) and mechanical ventilation (VIF = 5.31), which were within acceptable limits.

**Table 7:** The performance of the model in the validation cohort.

	AIS	s-ICH	SAH	TBI
AUC	0.72	0.82	0.76	0.73
Accuracy	0.91	0.91	0.68	0.70
Precision	0.94	0.91	0.73	0.71
Recall	0.96	1	0.75	0.91
F1	0.95	0.95	0.74	0.80

## Data Availability

Data are unavailable due to privacy or ethical restrictions.
